# Baseline predictors for good visual gains after anti-vascular endothelial growth factor therapy for myopic choroidal neovascularization

**DOI:** 10.1038/s41598-022-10961-y

**Published:** 2022-04-26

**Authors:** Cherng-Ru Hsu, Tso-Ting Lai, Yi-Ting Hsieh, Tzyy-Chang Ho, Chung-May Yang, Chang-Hao Yang

**Affiliations:** 1 Department of Ophthalmology, National Taiwan University Hospital, No.7, Chung-Shan South Rd, Taipei, Taiwan; 2 Department of Ophthalmology, Tri-Service General Hospital, National Defense Medical Center, Taipei, Taiwan; 3 Department of Ophthalmology, National Taiwan University College of Medicine, Taipei, Taiwan; 4 Department of Medical Science, Graduate School of National Defense Medical Center, Taipei, Taiwan

**Keywords:** Predictive markers, Retinal diseases

## Abstract

To investigate optical coherence tomography (OCT) and OCT angiography (OCTA) biomarkers for good visual outcomes in eyes with myopic choroidal neovascularization (mCNV) following anti-vascular endothelial growth factor (anti-VEGF) therapy. Patients diagnosed with mCNV via multimodal imaging were retrospectively reviewed. Baseline demographic data and biomarkers were collected. Anti-VEGF treatment based on a pro re nata (PRN) regimen was conducted on all eyes. The visual gains of ≥ 15 ETDRS letters or < 15 letters at 12-month were classified into two groups. Regression analysis was used to identify variables associated with significant best-corrected visual acuity (BCVA) improvement. Among 34 patients, 17 eyes and 17 eyes were classified into the two groups. There were no statistically significant differences in qualitative OCTA biomarkers between the two groups. The ≥ 15 letters group had significantly thicker subfoveal choroid thickness (SFCT) (79.97 ± 33.15 vs. 50.66 ± 18.31, P = 0.003), more ellipsoid zone integrity (58.8% vs. 23.5%, P = 0.037) and lower levels of fractal dimension (1.45 ± 0.101 vs. 1.53 ± 0.082, P = 0.031) than the < 15 letters group. SFCT and the ellipsoid zone integrity were correlated with 15 letters or more VA improvement in both univariable and multivariable analyses (P = 0.023 and P = 0.044, respectively). Thicker SFCT and integrity of the ellipsoid zone at baseline were associated with greater visual gains at 12 months. OCTA biomarkers seem to play a less important role in predicting the visual outcome of mCNV.

## Introduction

Myopic choroidal neovascularization (mCNV) is a vision-threatening complication in eyes with pathologic myopia and is often associated with poor prognosis if left untreated^1^. Currently, the gold standard treatment for mCNV is intravitreal anti-vascular endothelial growth factor (anti-VEGF) therapy^2^. A single injection followed by pro re nata (PRN) regimen is recommended^3^. In the REPAIR study, 86% of patients showed BCVA improvement, with 36.9% of patients achieving a BCVA gain of ≥ 15 ETDRS letters. The mean number of injections over 12 months was 3.6^4^. In the MYRROR study, a limited number of intravitreal injections could attain clinically important anatomical and visual benefits in the majority of patients^5^. The post-hoc analysis further showed that dosing frequency, visual acuity gains, and morphological outcomes were not influenced by baseline myopic macular degeneration severity^6^.

Novel imaging technologies, including spectral-domain optical coherence tomography (SD-OCT) and OCT angiography (OCTA), have increasingly been used to evaluate CNV characteristics. In contrast to wet age-related macular degeneration (wAMD), hyper-reflective material is located above the retinal pigment epithelium (RPE) and is usually accompanied by minimal exudative signs. The size of the lesion, morphology of the CNV margin, ellipsoid zone disruption, intra/subretinal fluid, and choroidal thickness have been reported with visual prognosis in previous studies^7–10^. Recently, perfusion of the retinal and choroidal vasculature and quantitative and qualitative features of the activity of the neovascular complex have been described in pathologic myopia^11,12^. Although different biomarkers for disease activity following intravitreal treatment have been mentioned, inconsistent consensus remains in the prediction of visual outcomes^13–15^. Therefore, the role of these imaging biomarkers in mCNV requires further research.

In the present study, we investigated both the structural OCT features and quantitative and qualitative OCTA biomarkers at baseline to identify the prognostic factors for prominent visual outcomes in naive mCNV eyes after anti-VEGF therapy within 12 months.

## Methods

This retrospective observational cohort study was performed by reviewing the medical records of patients who were observed at National Taiwan University Hospital between August 2018 and October 2020. This study was approved by the ethics committee of National Taiwan University Hospital and conducted according to the tenets of the Declaration of Helsinki.

### Study population

The initial diagnosis of mCNV was established using fluorescein angiography (FA) and SD-OCT. The inclusion criteria were as follows: (1) high myopia (axial length ≥ 26.5 mm and/or spherical equivalent refractive errors of ≤  − 6.0 diopters (D)); (2) fundus abnormalities consistent with the definition by the International Photographic Classification and Grading System for Myopic Maculopathy^16^; (3) a hyperfluorescent CNV network with an active leakage of fluorescein dye documented by FA; (4) presence of a hyperreflective component above the retinal pigment epithelium with overlying fuzzy areas and exudative signs on SD-OCT; and (5) treatment-naive eyes without any treatment history for myopic CNV, including laser, photodynamic therapy, or anti-VEGF. We reviewed medical records on 177 eyes of 176 consecutive patients with mCNV, and 143 eyes were excluded with the criteria. The exclusion criteria were (1) presence of other secondary choroidal neovascular diseases, such as wAMD, angioid streaks, or choroiditis (5 eyes); (2) combined vitreoretinal diseases, such as macular hole, foveoschisis, or retinal detachment with macular involvement (6 eyes); (3) poor FA or OCT image quality due to posterior staphyloma, media opacities or large amount of fundus hemorrhage (5 eyes); (4) poor quality of OCTA imaging (scan image quality index < 4/10) due to blinking artifacts, severe motion, projection artifacts or shadowing (121 eyes); (5) cataract surgery or other intraocular surgery during the follow-up period (4 eyes); and (6) the presence of advanced glaucoma, dense cataract, or amblyopia (2 eyes).

The treatment protocol and follow-up period received for patients in this study were in accordance with the PRN regimen in a previous report^1^, either using aflibercept (Eylea; Bayer, Leverkussen, Germany) or ranibizumab (Lucentis; Novartis, Basel, Switzerland) as single intravitreal anti-VEGF injection followed by additional injections as needed. If the visual acuity dropped by ≥ 5 ETDRS letters or signs of active disease occurred on OCT (e.g., intraretinal or subretinal fluid), then additional treatment was prescribed. Visual acuity stabilization was defined as no change in best-corrected visual acuity (BCVA) as compared with 2 preceding monthly visits. Treatment was stopped if the stabilization of visual acuity was achieved.

The demographic data of the patients, including age, sex, BCVA at baseline and at 12-month, ocular medical history, and number of intravitreal anti-VEGF injections were recorded. Clinically significant VA improvement was defined as ≥ 15 ETDRS letters at 12-month. The axial length (AL) was measured using a Lenstar LS 900 (Haag-Streit, Koeniz, Switzerland). Color fundus photography (CR-DGi Image Viewer; Canon Inc., Tokyo, Japan), FA, SD-OCT (RTVue RT-100, version 3.5; Optovue, Inc., Fremont, CA, USA), and OCTA (Optovue RTVue XR Avanti) were performed before the first anti-VEGF therapy in each patient. The image analysis was reviewed by two independent retina specialists blinded to the study groups. Any disagreements existed between interpretations were adjudicated by a third senior retina specialist.

### Image analysis acquisition

SD-OCT imaging of standard 10-mm horizontal and vertical scans centered on the fovea were obtained. Subfoveal choroidal thickness (SFCT) was measured as the distance from the outer border of the RPE at the point of the thinnest inner retinal layers on as a foveal point to the inner surface of the sclera using a manual caliper function of the built-in software. For eyes with disorganized retinal layers that were difficult to locate fovea, the central point of the foveal avascular zone (FAZ) on *en face* OCTA images was used to determine the foveal center. Central foveal thickness (CFT) was manually measured using the software. The integrity of the ellipsoid zone and the external limiting membrane (ELM), and presence of subretinal fluid were assessed within the central 1 mm. ELM and ellipsoid zone integrity were measured as the average of the horizontal and vertical OCT scans and graded as follows: 0 (intact or mild disruption < 1/2 within the central 1 mm) or 1 (severe disruption > 1/2 within the central 1 mm).

OCTA scanning area of 3 × 3 mm macula cube was obtained at baseline using the AngioVue System via split-spectrum amplitude-decorrelation angiography algorithm. The device was operated to produce two OCT volumes consisting of 304 × 304 A-scans each in approximately 2.6 s at a rate of 70,000 A-scans per second. *En face* images of the neovascular complex were obtained through automatic segmentation function in the OCTA built-in software from the RPE level to the outer border of Bruch’s membrane. For images with segmentation errors, the two horizontal boundaries of slab thickness were corrected according to B-scan information to visualize a clearer view of the neovascular complex. Either the choriocapillaris or the outer retinal slab was individually adjusted to include the entire CNV best.

Qualitative biomarkers of the CNV complex on OCTA were classified based on previous studies^11,17^: (1) morphological pattern, a medusa or seafan-shape versus tangled shape, (2) presence of tiny branching capillaries, (3) presence of anastomotic arcade and loops at the lesion periphery, and (4) presence of perilesional hypointense halo. The *en face* OCTA images were processed and analyzed using Fiji software (ImageJ; National Institutes of Health, Bethesda, MD, USA)^18^ for the CNV biomarkers. The angiography image size was first corrected for magnification considering AL-related parameters^19^. The CNV complex was then cropped and binarized using Otsu’s method for auto-thresholding. The skeletonized image was created according to the skeletonization function. Vessel density was calculated as the area occupied by neovascularization divided by the total area of the CNV complex after binarization. The vessel length density (VLD) value was determined by the ratio of the area occupied by skeletonized vessels (white pixels) after skeletonization^13,20^. Vessel diameter was calculating from vessel density divided by the VLD. Vessel tortuosity was measured as the actual length of each branch divided by the imaginary straight length between the two branch nodes^21^. Fractal dimension and lacunarity were calculated using the box-counting method, which are indices of morphological complexity and lesion inhomogeneity, respectively^22^.

### Statistical analysis

Statistical analysis was performed by SPSS software version 26 (SPSS Inc., Chicago, IL, USA). For descriptive statistics, means and standard deviations were used to present continuous variables, while frequencies and percentages were used to present categorical variables. Inter-group comparisons were performed using the chi-square test for categorical variables, while the Mann–Whitney U test was used for continuous variables. Logistic regression analysis was performed to identify biomarkers associated with greater visual gains. Variables with P < 0.1 during univariable analysis were included in the multivariable logistic regression. Linear regression analysis was used to examine the association between baseline SFCT and changes in BCVA. The threshold for statistical significance was set at P < 0.05.

## Results

### Demographic data and clinical characteristics

Thirty-four eyes from 34 patients with mCNV met the inclusion criteria for analysis in current study. The mean age of our cohort was 57.79 ± 17.26, and 30 (88.2%) were female. The mean AL was 29.01 ± 1.27 mm, and the mean spherical refractive error was − 12.18 ± 3.40 D. The mean baseline BCVA and the mean 12-month BCVA were 51.32 ± 20.20 and 60.59 ± 23.09 ETDRS letters, respectively. The mean SFCT was 65.32 ± 30.28 μm, and the mean CFT was 239.51 ± 105.05 μm at baseline. Seventeen eyes were classified into the “BCVA improved (≥ 15 letters)” group, whereas 17 eyes were categorized as the “BCVA maintained or worse (< 15 letters)” group.

**Figure 1:**
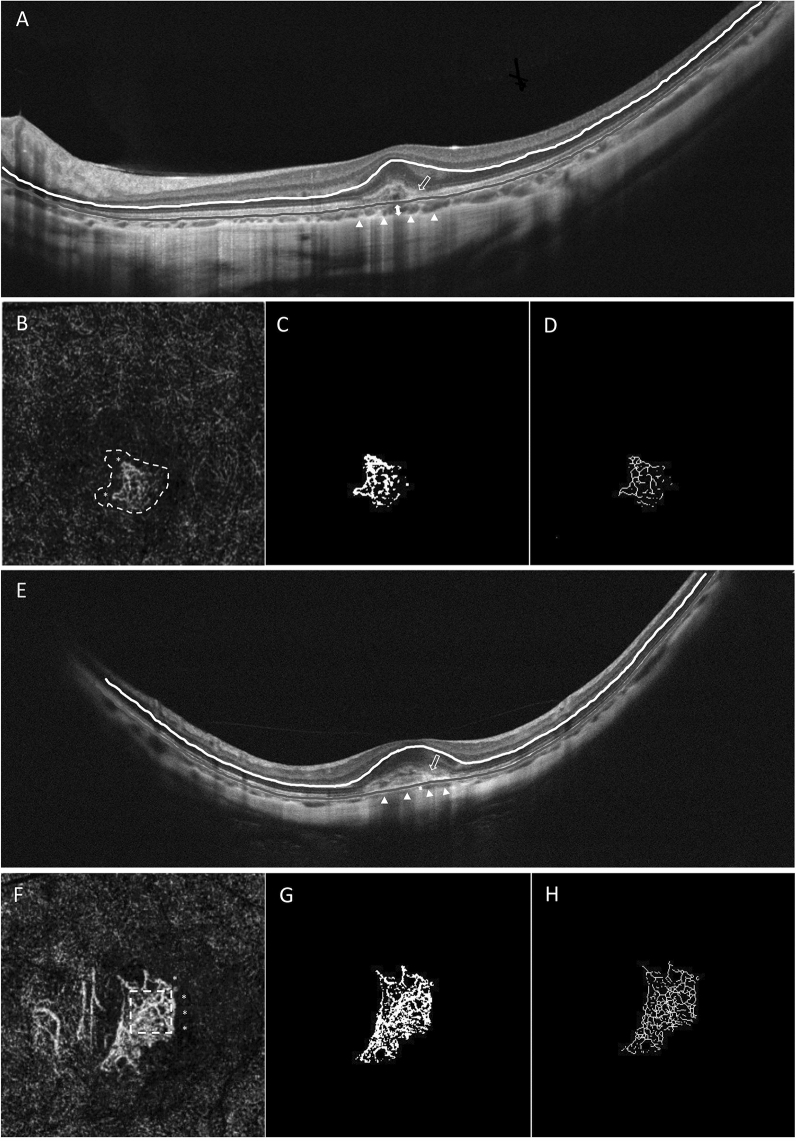
Baseline optical coherence tomography (OCT) and OCT angiography (OCTA) images of two patients with myopic choroidal neovascularization (mCNV). (**A**) This case is a 24-year-old female with an axial length of 29.20 mm. Baseline spectral-domain OCT B-scan shows a subfoveal choroidal thickness (SFCT) of 78.88 μm (double arrow). The choroid-scleral interface is indicated by white arrowheads. An ellipsoid zone disruption (graded 0) is found in the fovea (hollow arrow). (**B**) OCTA en-face image (3 × 3 mm) at baseline reveals a tangled-shaped NV with peripheral arcade/loops (*) and perilesional halo (white dashed line). The binarized and skeletonized image of the total NV lesion are showed in (**C**) and (**D**). Her baseline best-corrected visual acuity (BCVA) was 65 ETDRS letters, and improved to 85 EDTRS letters at 12 months after receiving one anti-vascular endothelial growth factor (anti-VEGF) injection. (**E**) This case is a 63-year-old female with axial length of 29.32 mm. Spectral-domain OCT B-scan at baseline shows an SFCT of 40.75 μm (double arrow). The choroid-scleral interface is indicated by white arrowheads. The absence of an ellipsoid zone (graded 1) is found in the fovea with haziness of the CNV border (hollow arrow). (**F**) OCTA en-face image (3 × 3 mm) at baseline shows a seafan-shaped NV with peripheral arcade/loops (*) and prominent branching vessels (white dashed square). The binarized and skeletonized image of the total NV lesion are showed in (**G**) and (**H**). Her BCVA was 70 ETDRS letters and 65 EDTRS letters at baseline and 12 months, respectively. She received a total of two anti-VEGF injections. The white lines in OCT B-scan represent the outer border of the outer plexiform layer, and the gray lines indicate the retinal pigment epithelium.

The baseline demographic data and clinical characteristics of the two groups are summarized in Table 1. There were no significant differences in age over 55 years old, sex, AL, spherical equivalent, or baseline BCVA. The baseline SFCT was significantly thicker in eyes with ≥ 15 letters improved group than eyes with < 15 letters group (79.97 ± 33.15 μm vs. 50.66 ± 18.31 μm, P = 0.003). No statistically significant difference was observed in the CFT between the two groups (P = 0.182). The ratio of eyes with grade 0 to grade 1 ellipsoid zone disruption was 10:7 in the ≥ 15 letters group and 4:13 in the < 15 letters group, respectively. A higher proportion of ellipsoid zone integrity was found in eyes belonging to the ≥ 15 letters improved group (58.8% vs. 23.5%, P = 0.037). The differences in ELM integrity and the presence of subretinal fluid were not significantly different between the two groups (P = 0.086 and P = 0.628, respectively). In *en face* OCTA analysis of the morphology of CNV lesions, a tangled pattern was exhibited in 11 eyes in the ≥ 15 letters group and in 13 eyes in the < 15 letters group (P = 0.452). There were no significant differences in OCTA qualitative biomarkers, including the presence of branching vessels (P = 0.486), anastomotic loops (P = 0.724), and perilesional dark halo (P = 0.300), between the two groups (Fig. 1). For quantitative biomarkers, significantly lower levels of fractal dimension were demonstrated in eyes in the ≥ 15 letters improved group (1.45 ± 0.101 vs. 1.53 ± 0.082, P = 0.031). Comparison of CNV size (P = 0.067), vessel density (P = 0.586), vessel length density (P = 0.946), vessel diameter (P = 0.812), vessel tortuosity (P = 0.683), and lacunarity (P = 0.454) did not differ significantly between the two groups. No difference existed between the two groups in the mean number of injections within 12 months (2.41 ± 1.23 vs. 2.76 ± 1.75, P = 0.683).

**Table 1:** Comparison of clinical characteristics between BCVA improved ≥ 15 letters and < 15 letters among choroidal neovascularization in myopic eyes.

	Eyes with BCVA improved (≥ 15 letters) (N = 17)	Eyes with BCVA maintained or worse (< 15 letters) (N = 17)	P value
Age (year), mean ± SD	53.71 ± 18.98	60.24 ± 15.73	0.243
Age ≥ 55 years, n	7	12	0.084
Sex (M vs F)	2/15	2/15	0.999
Axial length (mm), mean ± SD	28.79 ± 1.59	29.19 ± 0.94	0.482
Spherical equivalent (D), mean ± SD	− 12.18 ± 3.10	− 12.18 ± 4.45	0.799
Baseline BCVA (ETDRS letters), mean ± SD	49.12 ± 21.08	53.53 ± 19.67	0.474
12 months BCVA (ETDRS letters), mean ± SD	72.35 ± 15.62	48.82 ± 23.69	0.001*
**Structural OCT biomarkers**			
Subfoveal choroidal thickness (μm), mean ± SD	79.97 ± 33.15	50.66 ± 18.31	0.003*
Central fovea thickness (μm), mean ± SD	267.33 ± 119.62	211.69 ± 82.52	0.182
Ellipsoid zone integrity, grade 0 vs grade 1, n^†^	10/7	4/13	0.037*
ELM integrity, grade 0 vs grade 1, n^†^	11/6	6/11	0.086
Subretinal fluid, n (%)	2 (11.8)	3 (17.7)	0.628
**OCTA biomarkers**			
Morphology			
Medusa or sea-fan, n (%)	6 (35.3)	4 (23.5)	0.452
Tangled, n (%)	11 (64.7)	13 (76.5)	
**Qualitative biomarkers**			
Branching vessels, n (%)	8 (47.1)	6 (35.3)	0.486
Anastomotic loops, n (%)	6 (35.3)	7 (41.2)	0.724
Dark halo, n (%)	11 (64.7)	8 (47.1)	0.300
**Quantitative biomarkers**			
CNV size (mm^2^)	0.329 ± 0.257	0.481 ± 0.234	0.067
Vessel density (%)	41.31 ± 13.23	43.69 ± 7.66	0.586
Vessel length density (%)	21.21 ± 8.18	21.75 ± 4.52	0.946
Vessel diameter	2.15 ± 0.96	2.12 ± 0.62	0.812
Vessel tortuosity	1.22 ± 0.062	1.21 ± 0.097	0.683
Fractal dimension	1.45 ± 0.101	1.53 ± 0.082	0.031*
Lacunarity	0.367 ± 0.066	0.391 ± 0.079	0.454
No. of anti-VEGF treatments, mean ± SD	2.41 ± 1.23	2.76 ± 1.75	0.683

anti-VEGF: anti- anti-vascular endothelial growth factor; BCVA: best corrected visual acuity; CNV: choroidal neovascularization; D: diopters; ELM: external limiting membrane; No.: numbers; OCT: optical coherence tomography; OCTA: OCT angiography; SD: standard deviation.

^†^Ellipsoid zone integrity and ELM integrity were evaluated as the means of the horizontal and vertical scans and graded as: 0 (intact or mild disruption < 1/2 within the central 1 mm), 1 (severe disruption > 1/2 within the central 1 mm).

*Statistically significant (P < 0.05).

### Prognostic factors associated with significant visual acuity improvement

**Figure 2:**
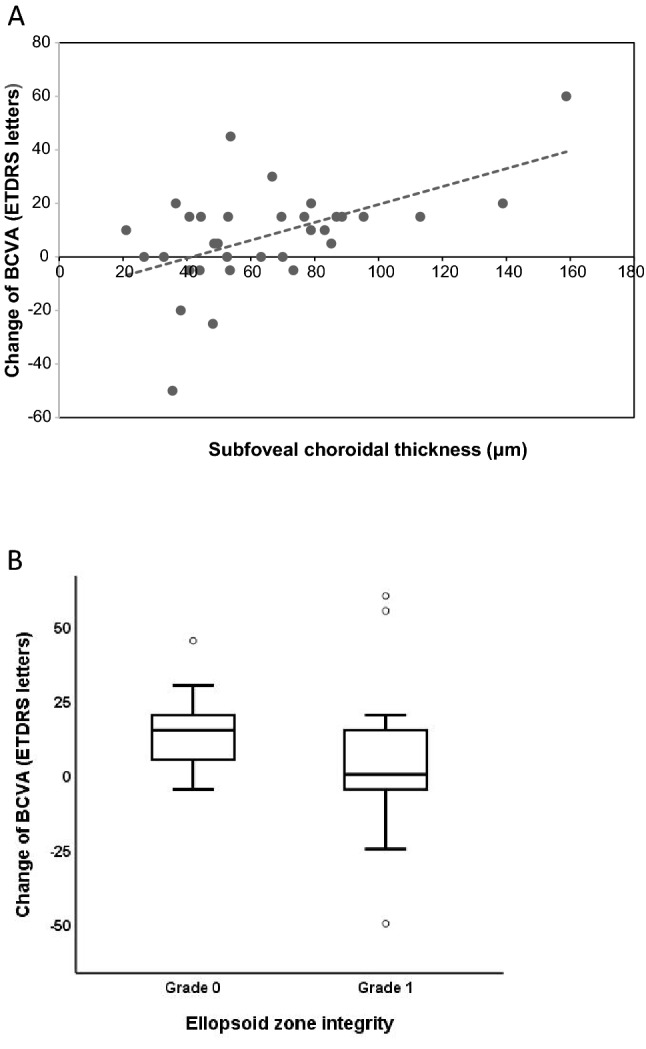
Analysis of the association between change in best-corrected visual acuity (BCVA) with subfoveal choroidal thickness (SFCT) and comparison change in BCVA with ellipsoid zone integrity. (**A**) The figure shows the linear regression and association analysis of SFCT with change in BCVA between baseline and 12 months (P = 0.002, r = 0.515). Dashed line represents the regression line. (**B**) Box plots of change in BCVA with ellipsoid zone integrity are shown. Quantitative analysis shows that there is significant difference in the grading of ellipsoid zone integrity (P = 0.049).

Univariable regression analysis revealed that eyes with BCVA that improved by ≥ 15 letters at 12 months were significantly associated with younger age, thicker SFCT, more intact ellipsoid zone integrity, and lower levels of fractal dimension at baseline (Table 2). None of the baseline qualitative OCTA biomarkers were statistically significant with greater visual gain. Multivariable regression analysis identified that only SCFT and ellipsoid zone integrity remained statistically significant with visual gain ≥ 15 letters at 12 months (P = 0.023 and P = 0.044, respectively) (Table 2). Linear regression analysis revealed that the change in BCVA was significantly associated with the baseline SFCT (P = 0.002, r = 0.515). Subgroup analysis showed that the change in BCVA differed significantly between the grades of ellipsoid zone integrity (P = 0.049) (Fig. 2).

**Table 2:** Univariable and multivariable logistic regression analysis of baseline biomarkers associated with significant visual acuity improvement at 12-month.

Variables	Univariable	P value	Multivariable	P value
	OR (95% CI)		OR (95% CI)	
Age	3.429 (0.827–14.209)	0.089	1.746 (0.139–21.873	0.666
Axial length (mm)	0.775 (0.424–1.419)	0.409		
Spherical equivalent (D)	0.999 (0.728–1.371)	0.994		
BCVA baseline	0.989 (0.955–1.023)	0.521		
**Structural OCT biomarkers**				
Subfoveal choroidal thickness (μm)	1.053 (1.012–1.096)	0.012*	1.076 (1.010–1.146)	0.023*
Central fovea thickness (μm)	1.006 (0.998–1.014)	0.141		
Ellipsoid zone integrity, (%)	4.643 (1.057–20.385)	0.042*	21.078 (1.092–406.842)	0.044*
ELM integrity, (%)	3.361 (0.823–13.722)	0.091	1.953 (0.258–14.777)	0.517
Subretinal fluid, (%)	1.607 (0.233–11.092)	0.630		
**OCTA biomarkers**				
Morphology				
Medusa or sea-fan/tangled, (%)	1.773 (0.396–7.932)	0.454		
**Qualitative biomarkers**				
Branching vessels, (%)	1.630 (0.411–6.459)	0.487		
Anastomotic loops, (%)	0.779 (0.195–3.118)	0.724		
Dark halo, (%)	2.062 (0.520–8.175)	0.303		
**Quantitative biomarkers**				
CNV size (mm^2^)	0.974 (0.945–1.004)	0.090	1.013 (0.962–1.065)	0.629
Vessel density (%)	0.979 (0.917–1.044)	0.513		
Vessel length density (%)	0.987 (0.888–1.096)	0.804		
Vessel diameter	1.054 (0.446–2.493)	0.905		
Vessel tortuosity	1.176 (0.001–1.252)	0.973		
Fractal dimension	0.915 (0.842–0.994)	0.036*	0.934 (0.833–1.046)	0.236
Lacunarity	0.953 (0.864–1.051)	0.334		
No. of anti-VEGF treatments	0.849 (0.553–1.351)	0.490		

anti-VEGF: anti- anti-vascular endothelial growth factor; BCVA: best corrected visual acuity; CI: confidence interval; CNV: choroidal neovascularization; ELM: external limiting membrane; D: diopters; No.: numbers; OCT: optical coherence tomography; OCTA: OCT angiography; OR: odds ratio.

*Statistically significant (P < 0.05).

## Discussion

In the present study, the associations between baseline structural and angiogenic biomarkers and the change in BCVA in naive mCNV eyes following anti-VEGF therapy were demonstrated. To the best of our knowledge, this is the first study to simultaneously illustrate OCT and OCTA variables for predicting significant visual improvement in patients with CNV due to myopia. OCT biomarkers including thicker SFCT and more intact ellipsoid zone integrity at baseline are significantly associated with gaining ≥ 3 lines of vision at 12 months, whereas initial quantitative and qualitative OCTA biomarkers are less sensitive in predicting significant visual improvements for mCNV. Furthermore, our study confirms the value of baseline SFCT by its positive association with visual outcome in eyes treated with anti-VEGF.

The imaging technique of OCTA enables the assessment of new qualitative features and detailed quantitation of microvascularity in CNV. In their study, Le et al.^15^ demonstrated that active neovascular complexes in myopic eyes were characterized by medusa or sea-fan-shaped vascular branching and the presence of loops/anastomosis in contrast to quiescent mCNV. In terms of morphology, previous research classified tangled mCNV patterns as low neovascular activity^12^. Most of the eyes (twenty-four out of 34) in our study were categorized into tangled shapes, which might reflect the nature of the low activity of mCNV itself. Moreover, the lack of statistically significant differences between the presence of branching capillaries, anastomotic loops, and a perilesional halo might suggest comparable initial CNV activity in both groups. The overall low injection numbers within 12 months between the two groups could also correspond to the low disease activity. In our study, the morphology pattern and qualitative biomarkers were not associated with clinically significant VA improvement at 12 months. For quantitative parameters, Hosoda et al.^13^ suggested that higher VLD and FD represented exuberant mCNV and were predictors of poor visual function. They also proposed that baseline VLD was correlated with treatment response in mCNV. Inconsistent with their findings, VLD did not correlate with visual outcomes in our study. Significantly higher levels of FD in the < 15 letters group indicated more initial vessel complexity, which may be attributed to the worse visual improvement. However, none of the OCTA biomarkers in our cohort remained statistically significant after multivariable analysis. Therefore, the utility of baseline OCTA biomarkers in predicting significant visual improvement might be clinically less important.

Thicker SFCT at baseline has been proposed as a “reserve” for more choroidal blood supply and better capacity for recovery in neovascular AMD compared to thinner SFCT, which often represents atrophic status^23^. In pathological myopia, progressive choroidal thinning leading to decreased choroidal perfusion might play an essential role in the development of myopic CNV and reduction in visual acuity^24^. The choroidal changes following anti-VEGF therapy reported by Ahn et al.^25^ observed that a thinner choroid at baseline was associated with incomplete resolution after a single anti-VEGF injection and with 1-year recurrence of mCNV, whereas SFCT, or its change, was not significantly associated with final BCVA. Another study suggested that thicker SFCT was a significant prognostic factor for final visual outcome^26^. Similarly, a thicker baseline SFCT remained significantly associated with ≥ 15 letters of BCVA gain at 12 months in the multivariable regression analysis in our cohort. In addition, thicker SFCT was positively correlated with greater BCVA gain in the linear regression analysis in our study. Taken together, we hypothesized that thicker SFCT might possess relatively abundant choroidal vascularity and more intact choriocapillaris responsive to achieving clinically meaningful VA gain after treatment, while eyes with thinner SFCT may suffer from choroidal ischemia and subsequent upregulation of angiogenic factors for mCNV recurrence.

Qualitative assessment of outer retinal integrity via measurement of the ellipsoid zone and ELM on OCT is potentially of significant value because their integrity has been regarded as a consistent biomarker for visual acuity^27^. Milani et al.^28^ inferred that the integrity of the ellipsoid zone at baseline was a positive predictive factor for final visual acuity after anti-VEGF treatment in patients with mCNV. Similarly, a better inner/outer segment line and ELM integrity showed a significant positive effect on BCVA outcome in a prospective study^14^. Since the fuzzy border and the hyperreflectivity of the neovascular complex could obscure the visibility and cause dilemma when judging on “present” or “absent” ellipsoid zone and ELM integrity on the OCT B-scan; therefore, we used a grading system to quantitatively assess the integrity. Less than half of the ellipsoid zone disruption at baseline was associated with greater BCVA gain compared to the more severe disruption in our study. Although lacking in statistical significance, more ELM integrity was observed in the group with ≥ 15 letters BCVA gain. Moreover, the change in BCVA showed a significant improvement in letters in grade 0 of the ellipsoid zone in our cohort, further confirming the positive association between visual outcome and ellipsoid zone integrity.

Although several studies have explored the predictors of visual outcome following treatment^29^, the diversity of results reflects the complicated pathophysiology of myopic CNV. With respect to age, Kuo et al.^30^ found that younger patients did not have a better outcome when compared to older patients. Guichard et al.^14^ showed that medium or higher baseline BCVA could predict a greater VA gain, while Holz et al.^31^ found a lower BCVA gain in patients with higher baseline BCVA. The discrepancy in thresholds for visual improvement may cause a significant effect on the association between baseline BCVA and visual outcome. We did not find any significant differences in baseline characteristics such as age above 55 years old, AL, sex, spherical equivalent, and BCVA in association with a final VA gain of more than three lines in our study.

The present study has several limitations, including its retrospective design and relatively small sample size. There was probably selection bias given that we excluded patients with a history of previous anti-VEGF treatment, low image signal quality, and inadequate follow-up duration. In terms of OCT image evaluation, only horizontal and vertical scans centered on the fovea were analyzed. To overcome the manual calibration error, we obtained the average of both scan measurements and graded the integrity of the ellipsoid zone and ELM, which allowed for a precise interpretation and lowered the observer-dependence. For OCTA analysis, the application of semiautomatic software for quantitative feature calculation could also help in interobserver concordance. In addition, OCT and OCTA images in the final visits were not included in our cohort to evaluate the serial changes in qualitative and quantitative biomarkers after treatment. Although we tried to investigate the baseline predictors that correlated with visual outcome, further studies with a larger number of subjects are required to elucidate the relationship between changes in VA such as the delay between symptoms and treatment, and changes in imaging parameters including grading of myopia maculopathy, or presence of a subretinal hemorrhage to clarify the role of ellipsoid zone integrity and SFCT.

In conclusion, the results of our study provide a detailed comparison of baseline OCT and OCTA characteristics that correspond to the visual outcome in mCNV lesions after anti-VEGF therapy. Structural OCT biomarkers, including thicker SFCT and integrity of the foveal ellipsoid zone at baseline, are associated with significant visual gains at 12 months. Qualitative and quantitative OCTA biomarkers seem to play a less important role in predicting visual improvements in myopic CNV.

## Data Availability

All data generated or analysed during this study are included in this published article.
